# KUAKINI HHP CENTER FOR TRANSLATIONAL RESEARCH ON AGING: LATEST FINDINGS FROM MODEL ORGANISMS TO HUMANS

**DOI:** 10.1093/geroni/igad104.0776

**Published:** 2023-12-21

**Authors:** Bradley Willcox, Richard Allsopp, Peter Martin

## Abstract

Kuakini Medical Center (Kuakini) was funded by NIH in late 2019 to create an interdisciplinary Hawai’i-based, Center of Biomedical Research Excellence (COBRE), for translational research on aging. This Center is building upon Kuakini’s five-decades of prior NIH-funded research. These resources include clinical data from the 58-year ongoing Kuakini Honolulu Heart Program (HHP), Honolulu-Asia Aging Study, HHP Offspring Study and a large biorepository with over 500,000 biological samples. The Center’s overarching aim is to increase infrastructure for collaborative aging research in Hawaii. The first step is to grow the Center’s faculty by hiring and mentoring research project leaders (RPLs) from diverse disciplines to become independent, R01-funded, investigators on aging. Our first RPL has graduated after obtaining R01-funded status. His project utilizes novel CRISPR methods to i) improve the safety and efficacy of delivering potentially therapeutic genes (such as FOXO3) to the mouse genome, and ii) test whether temporal enhancement of FOXO3 expression improves healthy aging in this mouse model - both key steps for potential translation to human clinical therapies. This work will be highlighted in the Program Overview session followed by current RPL findings. These findings include novel findings on a potential relation between FOXO3 genotype and stroke dynamics in elderly Japanese-American males; venous congestion in the brain and its potential impact on cognitive function; new findings on the Okinawan longevity phenomenon, among other interesting findings related to healthy aging. Supported by NIGMS 5P20GM125526 and NIA R01AG027060.

